# Photoperiod Genes Contribute to Daylength-Sensing and Breeding in Rice

**DOI:** 10.3390/plants12040899

**Published:** 2023-02-16

**Authors:** Leilei Qiu, Peng Zhou, Hao Wang, Cheng Zhang, Chengxing Du, Shujun Tian, Qinqin Wu, Litian Wei, Xiaoying Wang, Yiming Zhou, Rongyu Huang, Xi Huang, Xinhao Ouyang

**Affiliations:** 1 Rice Research Institute, Fujian Academy of Agricultural Sciences, Fuzhou 350002, China; 2 State Key Laboratory of Cellular Stress Biology, School of Life Sciences, Xiamen University, Xiamen 361102, China; 3 Rice Research Institute, Sichuan Agricultural University, Chengdu 611130, China; 4 Liaoning Rice Research Institute, Shenyang 110101, China

**Keywords:** photoperiod genes, daylength-sensing processes, latitude adaptation, rice breeding

## Abstract

Rice (*Oryza sativa* L.), one of the most important food crops worldwide, is a facultative short-day (SD) plant in which flowering is modulated by seasonal and temperature cues. The photoperiodic molecular network is the core network for regulating flowering in rice, and is composed of photoreceptors, a circadian clock, a photoperiodic flowering core module, and florigen genes. The Hd1-DTH8-Ghd7-PRR37 module, a photoperiodic flowering core module, improves the latitude adaptation through mediating the multiple daylength-sensing processes in rice. However, how the other photoperiod-related genes regulate daylength-sensing and latitude adaptation remains largely unknown. Here, we determined that mutations in the photoreceptor and circadian clock genes can generate different daylength-sensing processes. Furthermore, we measured the yield-related traits in various mutants, including the main panicle length, grains per panicle, seed-setting rate, hundred-grain weight, and yield per panicle. Our results showed that the *prr37*, *elf3-1* and *ehd1* mutants can change the daylength-sensing processes and exhibit longer main panicle lengths and more grains per panicle. Hence, the *PRR37*, *ELF3-1* and *Ehd1* locus has excellent potential for latitude adaptation and production improvement in rice breeding. In summary, this study systematically explored how vital elements of the photoperiod network regulate daylength sensing and yield traits, providing critical information for their breeding applications.

## 1. Introduction

The initiation of flowering, when plants transition from vegetative to reproductive growth, is one of the most crucial developmental decisions of the plant life cycle [1,2]. Flowering time is determined by a combination of endogenous genetic components and external environmental factors, such as photoperiod (daylength) and temperature [2]. Photoperiod induces flowering through three steps: the perception of the light signal, the regulation of the circadian clock, and flowering initiation [1,3].

Plants sense external light signals through different photoreceptors and transmit these light signals to the downstream circadian oscillator, which consists of a series of transcription−translation feedback loops, including the morning-phased proteins CCA1 (CIRCADIAN CLOCK ASSOCIATED 1), LHY (LATE ELONGATED HYPOCOTYL), RVE8 (REVEILLE 8), LNK1 (NIGHT LIGHT-INDUCIBLE AND CLOCK-REGULATED GENE 1), LNK2, PRR9 (PSEUDO-RESPONSE REGULATOR 9), and PRR7, and the evening-phased genes TOC1 (TIMING OF CAB2 EXPRESSON 1), ELF3 (EARLY FLOWERING 3), ELF4, LUX (LUX ARHYTHMO), and GI (GIGANTEA) [4,5]. The evening complex (EC) is composed of ELF3, ELF4, and the DNA-binding protein LUX, which together form a transcriptional repressor complex and a core component of the circadian oscillator [5]. LUX can bind directly to LUX-binding sites (LBS, GAT(A/T)CG) in the promoters of target genes and then recruit the EC to inhibit their transcription [5,6]. Mutation in any component of the EC results in phenotypic changes. The GI-CONSTANS (CO)-FLOWERING LOCUS T (FT) pathway is the main pathway involved in photoperiodic flowering in Arabidopsis (*Arabidopsis thaliana*) [1]. GI is degraded upon the formation of ELF3-CONSTITUTIVELY PHOTOMORPHOGENIC 1 (COP1)-GI complexes, which leads to a decrease in the expression of the flowering-promoting genes *CO* and *FT* [7]. Notably, *elf4* and *lux* mutants exhibit the same early-flowering phenotype as *elf3* mutants, suggesting that the EC complex suppresses flowering in Arabidopsis [8,9]. In addition, the other clock factors, CCA1 and LHY, can suppress the expression of *ELF3*, *ELF4*, and *LUX* by recognizing the so-called CCA1-binding site (CBS, AA(A/C)AATCT) in the *ELF3* promoter or the evening element (EE, AAAATATCT) in the *ELF4* and *LUX* promoters [5,9,10,11]. The circadian clock-related components show a circadian pattern of expression; in addition to regulating photoperiodic flowering, they also regulate plant growth and development via their involvement in plant stomatal movements, plant immunity, abiotic stress responses, photomorphogenesis, and aging [4,11,12,13,14].

As a typical short-day (SD) plant, rice exhibits critical daylength sensing with a threshold of 13.5 h [15]; rice flowers earlier under SD than under long-day (LD) conditions [16]. However, daylength in summer is always longer than 13.5 h in most rice-growing regions, particularly at high latitudes [16,17].

Rice employs a complex photoperiodic flowering network to control the expression of the florigen genes *Heading date 3a* (*Hd3a*) and *RICE FLOWERING LOCUS T 1* (*RFT1*) [18]. Florigen, the downstream integrator of the photoperiodic pathway, is translated in leaves and transported to the shoot apical meristem through the vasculature [18,19]. Hd3a and RFT1 form the flower-activating complex with 14-3-3 and FD, promoting the expression of the flowering-related genes, *OsMASD14* and *OsMADS15* [18,19]. The Hd1-Days to heading 8 (DTH8)-Grain number, plant height, and heading date 7 (Ghd7)-PRR37 module confers the critical daylength sensing in rice and modulates the expression of florigen genes under various daylengths [16]. Hd1, an ortholog of Arabidopsis CO, promotes flowering under SD conditions and represses flowering under LD conditions by regulating the expression of *Hd3a* and *RFT1* [17,20]. Therefore, to prevent the exposure of rice plants to low temperatures during the filling period at high latitudes, the loss-of-function allele *hd1* has been widely selected in rice breeding [21,22]. *DTH8*, encoding nuclear factor subunit YB-11 (NFYB-11), delays flowering under LD, and its natural variation allele *dth8* is common among cultivars [16,21,23]. *Hd1hd1 dth8dth8* and *hd1hd1 DTH8dth8* are the two major genotypes of *indica* hybrid rice that improve latitudinal adaptation in East Asia [16]. Shanyou63 (SY63), an outstanding hybrid rice variety in China because of its high yield and widespread geographical distribution, harbors the *Hd1hd1 dth8dth8* genotypic module, is associated with gradual daylength-sensing and underlies the molecular basis of the wide adaptability to photoperiod exhibited by this variety [24]. Ghd7 plays a crucial role in flowering by repressing the expression of *Ehd1*, *Hd3a*, and *RFT1* [16,25]. *Ghd7* mRNA levels increase with longer daylength, and Ghd7 loss-of-function or weak alleles lead to diminished photoperiod sensitivity (PS) in rice, underscoring the importance of *Ghd7* as a crucial gene for rice adaptation to high latitude [16,21,25].

The circadian clock, known as an endogenous timekeeping system, plays a major role in controlling plant growth and development. In recent years, many circadian clock genes have been cloned in rice. OsCCA1 (also named OsLHY) can directly bind to the CBS in the *OsGI* promoter to regulate the circadian rhythmic expression of *OsGI*, and then inhibits the transcription of *Hd1* in both LD and SD conditions [26]. When the expression of *OsCCA1* was downregulated, the number of tillers increased, the plants shortened, and the plants produced fewer panicles, with smaller grains [27]. In addition, OsCCA1 can directly bind to the promoter region of *Hd3a* to induce rice flowering under low nitrogen conditions [28]. The OsEC complex (OsELF4s-OsELF3-1-OsLUX) regulates flowering by binding to the *Hd1* and *Ghd7* promoters to repress their transcription [29]. ELF3 is a flowering repressor in Arabidopsis, but OsELF3-1 promotes rice flowering by reducing the expression of *Ghd7* [30]. A defect in *OsLUX* causes extremely late flowering and lower yields, while *Oself4-2* mutants flower late under LD conditions, indicating that OsEC is required for the circadian clock to regulate the flowering time in rice [29,31]. The *OsPRR* gene family, encoding core components of the circadian clock, plays an important role in regulating photoperiodic flowering in rice [18,32,33,34,35]. OsPRR37 delays flowering by negatively regulating *Ehd1* and *Hd3a* expression under LD conditions [33,34]. Knocking out *OsPRR73* led to early flowering under LD, but no change under SD conditions [35]. At the same time, the grain size and yield of the *Osprr73* mutant were significantly reduced under salt stress conditions due to the lower salt tolerance displayed by this mutant [36].

Natural variation in core flowering regulatory genes is widely used in rice breeding, while photoreceptor genes and circadian clock-related genes have rarely been exploited. This study demonstrates that mutations in the photoreceptor and circadian clock genes generate different daylength-sensing processes. The *se5* mutant, because it lacks active phytochromes, is deficient in photoperiodic responses and exhibits an early flowering phenotype and lower yield than the wild type. Mutation in *ELF3-1* and *Ehd1* can change the daylength-sensing processes and exhibit longer main panicle lengths and more grains per panicle. In addition, we identified single nucleotide polymorphisms (SNPs) or insertion/deletions (InDels) that introduced frameshifts, or large fragment deletions, in *Hd1*, *DTH8*, *Ghd7*, and *PRR37*. By contrast, we detected no frameshifts or InDel polymorphisms in *SE5*, *OsGI*, *Ehd1*, or *ELF3-1* among 115 rice germplasms. Collectively, these findings provide critical information for breeding applications of photoperiod genes.

## 2. Results

### 2.1. Photoperiod Genes Alter Daylength-Sensing in Rice

**Figure 1:**
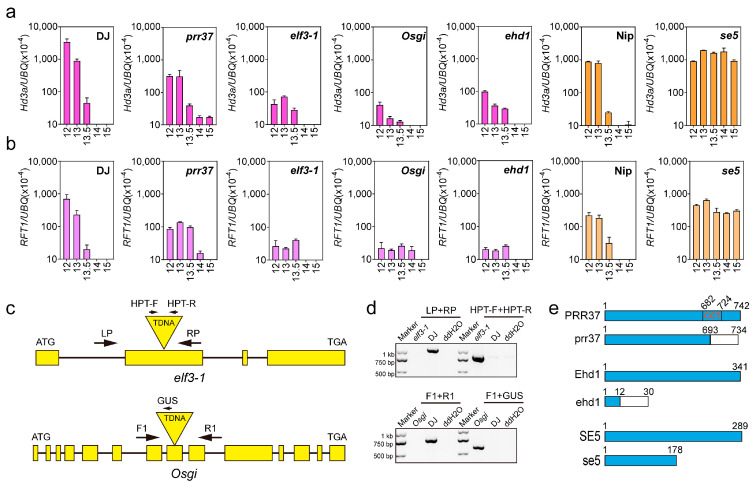
Using DEAS to detect rice daylength−sensing processes. (**a**,**b**) *Hd3a* (**a**) and *RFT1* (**b**) mRNA level under various daylength conditions. Relative mRNA levels determined by RT-qPCR. Wild-type plants (DJ and Nip) and mutants are indicated at the top of each pane. Numbers of hours of light in 24 h light−dark cycles are indicated on the *x* axis. Error bars represent the standard deviation (S.D.) of three independent RT-qPCR measurements. (**c**) Diagram of *OsELF3-1* and *OsGI* gene structure and the T-DNA insertion site. The yellow filled boxes indicate exons, and the solid lines represent introns. Arrows indicate the primers used for genotyping the insertion site. (**d**) PCR genotyping of *elf3-1* and *Osgi* mutants. (**e**) The blue rectangles show the lengths of PRR37, Ehd1, and SE5 proteins in number of amino acids. The white rectangles indicate frameshift mutations in PRR37, Ehd1, or SE5.

In our previous study, we developed a daylength-sensing-based environment adaptation simulator (DEAS) to forecast rice latitude adaptation via the transcriptional dynamics of florigen genes at different latitudes [16]. To assess whether loss-of-function alleles in photoperiod genes might affect daylength sensing, we measured the expression levels of *Hd3a* and *RFT1* under various daylengths (daylength-sensing processes) in the rice cultivar Dongjin (DJ, wild type), Nipponbare (Nip, wild type), as well as the mutants *Osgi*, *prr37*, and *elf3-1*, grown under various daylengths (DEAS step 1). The DJ and Nip seedlings sensed a critical daylength (threshold = 13.5 h), as the expression of *Hd3a* and *RFT1* was only induced when the daylength fell below 13.5 h (Figure 1a,b). In the *prr37* mutant, the *Hd3a* expression was lower than in the DJ under daylengths shorter than 13.5 h, but higher for daylengths longer than 13.5 h (Figure 1a). *RFT1* expression was also lower than that of DJ at photoperiods shorter than 13 h in *prr37* mutant, but was higher at 13.5 h and 14 h daylengths, and became undetectable in the DJ and the *prr37* mutant under a 15 h daylength (Figure 1b). The *prr37* mutant exhibited gradual daylength sensing for *Hd3a* expression and critical daylength sensing (threshold = 15 h) for *RFT1* expression. Compared to DJ, the *Hd3a* and *RFT1* expression was lower in *elf3-1* when daylength was shorter than 13.5 h, but comparably lower in DJ and *elf3-1* when the daylength was longer than 14 h. The *elf3-1* mutant sensed a fine-tuned critical daylength threshold (14 h). However, the expression of *RFT1* in the *Osgi* mutant was higher than that of *elf3-1* under a 14 h daylength, and the expression of *Hd3a* and *RFT1* was broadly similar in the *Osgi* and *elf3-1* seedlings at the other daylengths tested (Figure 1a,b). The *Osgi* mutant exhibited critical daylength sensing for *Hd3a* and *RFT1* expression, and the threshold was 14 and 15 h, respectively. These results indicate that mutations in circadian clock genes lead to changes in the daylength-sensing, to varying degrees, in rice.

Ehd1 is a hub component in rice photoperiodic flowering and promotes flowering in both LD and SD conditions [18,37]. We determined that the *ehd1* and *elf3-1* mutants exhibit the same daylength-sensing processes in DEAS (Figure 1a,b). The first step of photoperiodic flowering is the perception of a light signal. *SE5* encodes a heme oxygenase that participates in the chromophore biosynthesis for the red/far-red light photoreceptors phytochromes (phys) [38]. In the *se5* mutant, *Hd3a* and *RFT1* were expressed at high levels regardless of the photoperiod (Figure 1a,b), as was previously reported for the *phyAphyB* and *phyAphyC* double mutants [39]. Taken together, these results suggest that mutations in the circadian clock genes and photoreceptor genes greatly affect the expression of *FT* orthologs (*Hd3a* or *RFT1*) when rice plants are grown under different daylengths.

### 2.2. The DEAS Couples Latitude Adaptation and Daylength Sensing

**Figure 2:**
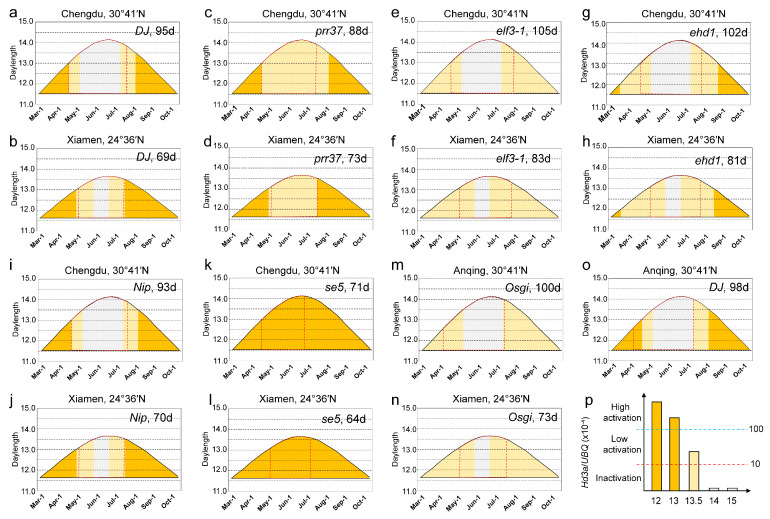
The effect of latitude and genotype on dynamics of *Hd3a* transcript levels. (**a**–**l**) Predicted *Hd3a* expression during the growing season in Xiamen (24°36′ N) and Chengdu (30°41′ N) and relation dynamics of *Hd3a* transcription in response to different latitudes to flowering time in DJ (**a**,**b**), *prr37* (**c**,**d**), *elf3-1* (**e**,**f**), *ehd1* (**g**,**h**), Nip (**i**,**j**) and *se5* (**k**,**l**). (**m**–**o**) Predicted *Hd3a* expression of *Osgi* (**m**,**n**) and DJ (**o**) in Xiamen (24°36′ N) and Anqing (30°41′ N). (**p**) The three levels of *Hd3a* gene-expression. The red dotted box represents the time from sowing to flowering. Flowering time is indicated at the top of each heatmap. Dark yellow, light yellow, and gray indicate high activation, low activation, and inactivation of *Hd3a* transcription, respectively.

The latitude adaptation means that crops adapt to a given latitude when they can complete their entire growth period in a specific ecological environment. To explore the relationship between latitude adaptation and daylength sensing, we planted DJ, *prr37*, *Osgi*, *elf3-1*, *ehd1*, Nip, and *se5* in regions with latitudes of 30°41′ N and 24°36′ N. We also planted DJ, *elf3-1*, *ehd1*, Nip, and *se5* at 41°40′ N. We measured the daylengths between March 1st and October 11th at three latitudes (Figure 2 and Figures S2 and S3). We previously established that, based on the expression of *Hd3a* (or *RFT1*) under various daylengths in DEAS step1, DEAS step 2 can use the daylength dynamics at a given latitude to infer an expression heatmap for *Hd3a* (or *RFT1*) at that latitude [16]. We thus produced expression heatmaps for *Hd3a* and *RFT1* in DJ, *prr37*, *Osgi*, *elf3-1*, *ehd1*, Nip, and *se5* at the two locations with latitudes of 30°41′ N and 24°36′ N (Figure 2 and Figure S2) using the measured expression levels of *Hd3a* and *RFT1* in DEAS step 1 (Figure 1a,b).

Notably, we predicted the transcriptional inactivation of *Hd3a* in DJ, *elf3-1*, *ehd1*, *Osgi*, and Nip (Figure 2). We found a longer period of transcriptional inactivation for *Hd3a* and a later flowering time at the higher latitude region (Figure 2). Compared with the planting in the 30°41′ N region, the flowering time of DJ, *prr37*, *elf3-1*, *ehd1*, and Nip in 24°36′ N was 26, 15, 22, 21, and 23 days earlier, respectively. Notably, PRR37 promoted rice flowering in the 24°36′ N region (Figure 2b,d) and repressed rice flowering in the 30°41′ N region (Figure 2a–d). At the same time, we predicted a high transcriptional activation of *Hd3a* and *RFT1* throughout the growth period in *se5*, but the flowering times were different among the three regions (Figure 2k,l and Figure S3d). We speculate that the daylength at the lower latitudes is shorter than at higher latitudes, and that the temperatures differed among the three regions. These results suggest that rice flowering time is related to the number of days of transcriptional inactivation, low transcriptional activation, and high transcriptional activation of *Hd3a* and *RFT1*.

### 2.3. Photoperiod Genes Affect Rice Yield

**Figure 3:**
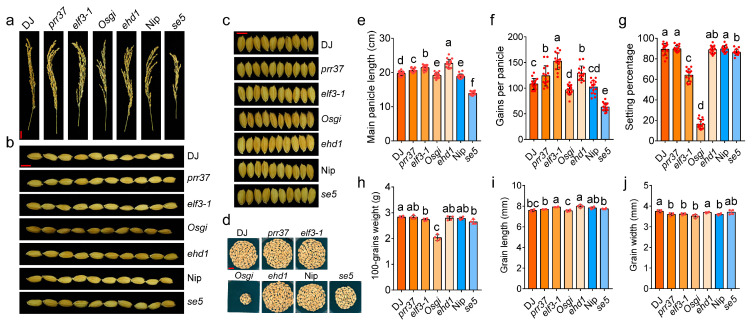
Phenotypic characterization of main panicles and grains of the wild-type and mutant. (**a**–**d**) The phenotypes of main panicle (**a**), grain length (**b**), grain width (**c**)**,** and yield per panicle (**d**) of DJ, *prr37*, *elf3-1*, *Osgi*, *ehd1*, Nip, and *se5* at the mature stage. The red lines are scale bars. (**e**–**j**) Main panicle length (**e**), grains per panicle (**f**), setting percentage (**g**), hundred-grain weight (**h**), grain length (**i**), and grain width (**j**) of DJ, *prr37*, *elf3-1*, *Osgi*, *ehd1*, Nip, and *se5*. Data are presented as means ± SD, n = 15 (**e**–**g**), or four biological replicates (**h**–**j**). Scale bars: 2 cm for main panicle; 5 mm for grain length; 5 mm for grain width; 1 cm for yield per panicle. The letters above each column indicate significant differences by Duncan’s multiple range test (*p* < 0.05). All agronomic traits of the wild-type and mutant were collected under natural conditions in Xiamen.

Agronomic traits, such as the number of grains per panicle, hundred-grain weight, and seed-setting rate, are closely related to rice yield [40]. In addition to flowering time, we wonder if photoperiod genes affect rice yield. To this end, we measured the main panicle length, number of grains per panicle, seed-setting rate, hundred-grain weight, grain length, grain width, and yield per panicle of DJ, *prr37*, *Osgi*, *elf3-1*, *ehd1*, Nip, and *se5* plants (Figure 3 and Figure S4). We observed that *prr37*, *elf3-1*, and *ehd1* mutants have longer growth periods, accompanied by longer main panicles and more grains per panicle (Figure 2d,f,h and Figure 3a,e,f). By contrast, the *se5* mutant had the shorter growth period, shorter main panicles, fewer grains per panicle, and lighter yield per panicle (Figure 2l, Figure 3a,e,f and Figure S4). Notably, mutations in the core circadian clock genes *ELF3-1* and *OsGI* led to lower setting rates (Figure 3g), particularly *OsGI*, as the *Osgi* mutant showed low values for the hundred-grain weight and yield per panicle (Figure 3h and Figure S4). In previous reports, the *Osgi* mutant was shown to display reduced fertility under atypical growing conditions with late transplanting dates [41]. Mutations in *PRR37*, *OsGI*, *ELF3-1*, *Ehd1*, and *SE5*, caused almost no changes in the grain length or grain width (Figure 3i,j). Importantly, the *Osgi* and *se5* mutants had a lower yield per panicle, and the *prr37* and *ehd1* mutants had higher yields per panicle (Figure 3d and Figure S4). Collectively, these results suggest that photoperiod genes not only change the daylength-sensing processes, but also the agronomic traits in rice.

### 2.4. Nucleotide Polymorphism of Photoperiod Genes in Rice cultivars

**Figure 4:**
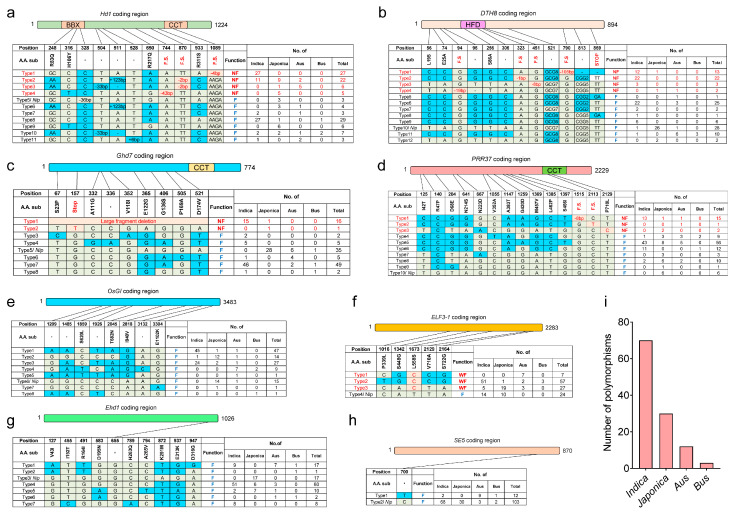
Haplotype analysis of *Hd1*, *DTH8*, *Ghd7*, *PRR37*, *OSGI*, *ELF3-1*, *Ehd1*, and *SE5* coding sequences in 115 rice germplasms. (**a**–**h**) The Hd1 (**a**), *DTH8* (**b**), *Ghd7* (**c**), *PRR37* (**d**), *OSGI* (**e**), *ELF3-1* (**f**), *Ehd1* (**g**) and *SE5* (**h**) nucleotide sequences were compared with that of Nip. Polymorphic nucleotides are indicated by different colors. NF, Non−functional; WF, Weak functional. The number of *indica*, *japonica*, *Aus*, and *Bus* with each genotype is shown in the chart at the right, with the numbers for NF and WF types in red. F.S., frame shift. (**i**) The number of *indica*, *japonica*, *Aus*, and *Bus* in 115 rice germplasms.

The Hd1-DTH8-Ghd7-PRR37 module regulates rice daylength-sensing in rice, and natural variation in the underlying photoperiod genes changes the daylength-sensing processes, which play a vital part in the adaptation of rice cultivars to multi-latitude regions [16,24]. To expand this analysis to photoperiod genes, we collected the DNA sequences for *Hd1*, *DTH8*, *Ghd7*, *PRR37*, *SE5, OsGI*, *Ehd1*, and *ELF3-1* from 115 rice germplasm resources (70 *indica*, 30 *japonica*, 12 *Aus*, and 3 *Bus*) [42,43,44,45,46,47,48,49,50,51], and looked for polymorphisms (Figure 4). Among the 115 rice materials, we identified 11 haplotypes for *Hd1*, of which seven (in 55 rice varieties) were functional, while the remaining four haplotypes (present in 60 rice varieties) were predicted to encode a non-functional protein due to the presence of frameshifts (Figure 4a). Likewise, we detected 12 haplotypes for *DTH8*, with 40 rice varieties harboring non-functional alleles, including 37 *indica* (Figure 4b). This result was consistent with our previous study, in which we determined that *Hd1hd1 dth8dth8* is one of the major genotypes of *indica* hybrid rice in East Asia [16]. We also observed two non-functional haplotypes at *Ghd7*, one with a large fragment deletion and the other with early translation termination, accounting for 14.8% (or 17) of all accessions among the 115 germplasms (Figure 4c). We identified three non-functional variants in *PRR37*, two with frameshift mutations and the one with three amino acid substitutions (Figure 4d). Three of them (N214S, L462P, and P710L) were located at the conserved positions among their homologs, and a previous study suggested that these substitutions may affect the PRR37 function [34].

Notably, we detected no frameshift mutations or deletions in the coding sequences of *OsGI*, *ELF3-1*, *Ehd1*, or *SE5* among the 115 rice germplasms (Figure 4e–h). *SE5* is highly conserved, without any amino acid variation in the 115 germplasms (Figure 4h). One amino acid substitution (G219R) in the Golden2, Arabidopsis RESPONSE REGULATOR (ARR), and Chlamydomonas regulatory protein of P-starvation acclimatization response (Psr1) (GARP) domain of Ehd1 was previously demonstrated to lower the DNA-binding activity of Ehd1 [37,52]. However, this Ehd1^G219R^ allele is rare and was not represented in our panel of 115 varieties (Figure 4g). ELF3-1 isoforms can be divided into ELF3-1(L) and ELF3-1(S) (weak function) based on the amino acid at position 558. Overall, 79.1% (or 91) of the 115 varieties produce ELF3-1(S) (Figure 4f). Compared to ELF3-1(L), ELF3-1(S) delayed rice flowering under LD conditions [53]. The *japonica* varieties carrying ELF3-1(L) occur at higher latitudes, while the varieties carrying ELF3-1(S) are found at lower latitudes [54].

## 3. Discussion

The timing of flowering is a key agronomic trait that determines the latitudinal adaptability and planting seasons of rice cultivars. A suitable flowering time allows rice plants to make full use of light and temperature resources to maximize yield [16,55]. *Indica* rice cultivars with the genotype *Hd1 DTH8 Ghd7 PRR37* are characterized by extremely low expression of florigen when the daylength is longer than 13 h, and fail to flower under natural LD conditions [24]. However, our results also indicate that the time to flowering of DJ and Nip (*japonica*, *Hd1 DTH8 Ghd7 PRR37* genotype) was about 145 days at 41°40′ N (Figure S3a), which exceeds the suitable growing season. The selection of rice varieties adapted to various latitudes has capitalized on the variations in the core flowering regulatory genes *Hd1*, *Ghd7*, *DTH8*, and *PRR37* [16,17,21,34]. A loss-of-function mutation of any of these four genes results in reduced PS, and different allelic combinations at these four genes exhibit diverse degrees of PS, ranging between strong PS and complete photoperiod insensitivity [16,17,21,55]. The wild rice *O. rufipogon* harbors functional alleles at *Hd1*, *DTH8*, *Ghd7*, and *PRR37* (*HDGP*) and thus possesses strong PS [55]. In rice, the natural variation of core photoperiod genes is an important molecular basis for latitudinal expansion. In this study, we found frameshift mutations or deletions in the coding sequences of *Hd1*, *DTH8*, *Ghd7*, and *PRR37* among some rice varieties (Figure 4). In addition, in the Heilongjiang Province of China (situated at relatively high latitudes), many local modern *japonica* varieties carry non-functional *hd1*/*ghd7*/*prr37* (*hgp*) and weak functional *DTH8* (or weak-functional *DTH8/Ghd7* and non-functional *hd1*/*prr37*) alleles, resulting in weak PS to match the lower temperature and longer daylength of the region [55]. Very few hybrid rice varieties carry strong functional alleles at *Hd1*/*DTH8*/*Ghd7*, because their combination produces strong PS and extremely late flowering time [55]. In addition to the four core flowering regulatory genes (Figure 4a–d), several minor genes are also used in breeding, such as *Hd16*, *DTH2*, *OsMADS56*, and *RFT1* [56,57,58,59].

Plants perceive light signals through various photoreceptors, such as the red/far-red light receptor phytochromes and blue light receptor cryptochromes [1]. There are three phytochromes in rice: OsPHYA, OsPHYB and OsPHYC. Under natural LD conditions, *phyB* and *phyC* single mutants show an early-flowering phenotype, while *phyA* does not change the flowering time [60]. The *phyAphyC* and *phyAphyB* double mutants flower very early under natural LDs and exhibit daylength insensitivity [39,60], similar to the daylength-sensing processes of the *se5* mutant (Figure 1a,b). Meanwhile, the seed setting rate of the *phyAphyB* double mutant is significantly decreased [61]. There are three cryptochromes in rice, OsCRY1a, OsCRY1b, and OsCRY2, but only OsCRY2 promotes flowering under both SD and LD conditions [62]. In addition, we revealed that the yield per panicle of the *Osgi* and *se5* mutants was significantly decreased compared to that in the wild type (Figure 3d and Figure S4). These findings indicate that photoreceptor genes and circadian clock genes can not only regulate daylength-sensing processes (Figure 1), thereby changing the flowering time and latitude adaptation, but also affect yields in rice (Figure 3 and Figure S4).

Soybean is a typical SD plant, and the suitable flowering time guarantees high yields. E1, a flowering repressor in soybean, is a hub of the photoperiodic flowering network, which represses the expression of the florigen genes, *GmFT2a* and *GmFT5a*, to delay flowering. E2 (GmGI), E3 (GmphyA3) and E4 (GmphyA2) repress flowering and induce the expression of E1. The natural variation of *E1*, *E3* and *E4* change the soybean daylength-sensing processes, expanding the planting area to higher latitudes [63,64,65]. The soybean *J* gene, as the ortholog of Arabidopsis *ELF3*, represses the expression of *E1* to promote soybean flowering. The *J* gene of soybean and the *ELF3-1* gene of rice function similarly, both as flowering activators (Figure 2a,b,e,f). Mutation of the soybean *J* locus prolongs the vegetative growth period and improves its adaptation to the tropics [66]. In the process of soybean domestication, the mutation in *Tof11* (*PRR3b*) and *Tof12* (*PRR3a*) contributed to flowering, and the plants matured earlier, improving the higher latitude adaptation [67]. Compared to wild soybean, the cultivars in Northeast China carry higher abundant non-functional *tof12* [67]. *Tof5* is also related to soybean higher latitude adaptation, which promotes flowering via inducing the expression of *FT2a* and *FT5a* [68]. The novel locus *Tof16* (a homolog of *LHY*) is a repressor of E1 and enabled the soybean to migrate from its temperate origin to the tropics; more than 80% of accessions in low-latitude areas contain loss-of function *tof16* and *j* [69]. Haplotypes of *GmFT2a* and *GmFT5a* are also involved in the soybean flowering phenotypes, maturity time and geographical distributions [65,70].

Maize was domesticated from teosinte (*Zea mays ssp*. *parviglumis*) and originated in southwestern Mexico [71]. *ZCN8* is homologous to Arabidopsis *FT*, the SNP-1245 and InDel-2339 in the *ZCN8* promoter regions play an important role in maize expanding to high latitudes [72]. ZmCCT9 and ZmCCT10 contain a CCT domain, homologous to rice Ghd7, repress the expression of *ZCN8*, and delay flowering under LD conditions [73,74]. The transposon insertion upstream of *ZmCCT9* and *ZmCCT10* reduces their expression and accelerates the process of maize adapting to higher latitudes [73,74].

There are various photoperiod genes involved in the latitudinal adaptation of rice, soybean, and maize. In rice and maize, transcription factors are widely selected in breeding, such as Hd1, DTH8, Ghd7 (Figure 4), ZmCCT9, and ZmCCT10. However, photoreceptors (*E3* and *E4*) and circadian clock genes (*Tof11*, *Tof12*, and *J*) are widely used in soybean breeding. In lower latitudes, local farmers prefer double or triple cropping rice throughout the year to harvest more grain. Mutations in *Ehd1* or *ELF3-1* prolong the growth period in rice, so they are probably not suitable for rice multiple cropping. Soybean from temperate regions introduced to lower latitudes flower early and have an extremely low yield. Natural variation at the soybean *J* (*ELF3*) locus extends the vegetative phase under inductive SD conditions and increases yield [66].

Diverse combinations of the *Hd1*, *DTH8*, *Ghd7*, and *PRR37* genes mediate the multiple daylength-sensing processes to improve the latitude adaptation [16]. However, optimal cropping modes coupled with proper daylength-sensing processes can enhance rice multi-latitude adaptation [24]. Mutations in some circadian clock genes and photoreceptor genes greatly affect the daylength-sensing processes and yield-related traits, resulting in a mismatch between the growth period of crops and the arable season. Notably, our results indicated that the *ehd1* and *elf3-1* mutants had longer main panicle lengths and more grains per panicle than DJ (Figure S4). Based on our findings and previous research [75,76], mutations in *Ehd1* and *ELF3-1* improved the grain and yield (Figure 3 and Figure S4). Hence, with global warming, as the growing season length will be extended, perhaps the *Ehd1* and *ELF3-1* locus could be applied to rice breeding in the future.

## 4. Materials and Methods

### 4.1. Plant Materials

Specific target sites were designed using an online toolbox (http://crispr.dbcls.jp/) [77]. For the *ehd1* mutant, three unique guide RNAs, 5′-ggcATCACTCACTGTCTTCTCCG-3′, 5′-gccGCCTTATGGACTAAGAGTTC-3′, and 5′-gttGCGTTCTTTCCTACCGAAGA-3′, were cloned into U3- (gRNA), U6a-gRNA, and U6b-gRNA respectively. For the *prr37* mutant, one guide RNA, 5′-gccgAAAAGGAAAGAGCGCAACTT-3′, was inserted into U6a-gRNA. These guide RNAs were cloned into the pYLCRISPR/Cas9-Mtmono vector and transformed into DJ callus. Rice plants were transformed by *Agrobacterium* strain EHA105.

The rice (*Oryza sativa*) subspecies japonica DJ and Nip were used as the wild type. The T-DNA insertion mutants *elf3-1* and *Osgi* were previously reported [30,78]. The *se5* mutant with a nucleotide mutation caused an early termination. All of the recombinant vectors and mutants were confirmed by PCR or sequencing. The DNA sequencing results are shown in Supplementary Figure S1. The primers are listed in Table S1.

### 4.2. Plant Growth Conditions

For the RT-qPCR assay, rice seedlings were grown in growth chambers with 28 °C and a relative humidity of ~70% for 35 days. Six growth chambers were used with 12, 13, 13.5, 14 and 15 h daylengths, respectively. The light in the growth chambers was supplied by light-emitting diodes (Sanan Sino-Science, Xiamen, China). For the flowering phenotypic assays in the field, seeds were sown in Xiamen (24°36′ N, 6 May 2022), Chengdu (30°41′ N, 18 April 2022), Anqing (30°41′ N, l7 Apri 2021) and Shenyang (41°40′ N, 20 April 2021).

### 4.3. Analysis of Gene Expression

On day 35, all of the rice seedling leaves were harvested 3 h after dawn. The total RNA was isolated from the samples with an Eastep Super Total RNA Extraction Kit (Promega, Beijing, China) and were reverse transcribed with the GoScript Reverse Transcription Mix using oligo(dT) (Promega, Beijing, China), according to the manufacturer’s instructions. Real-time quantitative PCR (RT-qPCR) was performed using the SYBR Green PCR (Bio-Rad) method on a CFX Connect^TM^ Real-Time PCR System (Bio-Rad, California, USA), following the manufacturer’s instructions. The transcriptional data for *Hd3a* and *RFT1* were then collected. When the expression of *Hd3a* and *RFT1* differed by more than 10-fold between adjacent daylengths, we categorized this expression pattern as critical daylength sensing. When the florigen gene expression changed gradually with the change in the daylength and the difference between the adjacent daylengths was less than 10-fold, we categorized this expression pattern as representing gradual daylength sensing. The primers used for the RT-qPCR are listed in Table S1.

### 4.4. Florigen Gene Expression Heatmap

First, we divided the expression of florigen genes into three levels in the inferred *Hd3a* and *RFT1* expression profiles. When the *Hd3a* or *RFT1* expression was <10 × 10^−4^, we defined them as being inactivated. When the 10 × 10^−4^ <*Hd3a* or *RFT1* expression <100 × 10^−4^, we defined them as being weakly inactivated. When the 100 × 10^−4^ <*Hd3a* or *RFT1* expression, we defined them as being highly inactivated. Subsequently, we obtained the florigen gene-expression heatmap according to the methods previously described [16,24]. The daylength data for different latitudes were collected using the Rise and Set Times app developed by S. Vdovenko (http://www.lifewaresolutions.com/ (accessed on 30 January 2020)).

## Data Availability

Data is contained within the article or supplementary material.

## Supplementary Materials

The following supporting information can be downloaded at: https://www.mdpi.com/article/10.3390/plants12040899/s1, Figure S1: Sequence of different mutant alleles., Figure S2: The effect of latitude and genotype on dynamics of *RFT1* transcript levels., Figure S3: The effect of latitude and genotype on dynamics of *Hd3a* transcript levels., Figure S4: Yield per panicle of wild-type and mutant; Table S1: The primers used in this study.
