# Supplementary material for: Photoperiod Genes Contribute to Daylength-Sensing and Breeding in Rice
Source: Plants (Basel). 2023 Feb 16;12(4):899. doi: 10.3390/plants12040899 (PMC9959395; doi:10.3390/plants12040899)
Supplement: Supplementary file 1 [file plants-12-00899-s001.zip › plants-2173096-supplementary.pdf]

plants

MDPI

Table S1. The primers used in this study.

Primer	Sequence (5′ to 3′)
**qRT PCR**	
qHd3a-F	GCTCACTATCATCATCCAGCATG
qHd3a-R	CCTTGCTCAGCTATTTAATTGCATAA
qRFT1-F	TGACCTAGATTCAAAGTCTAATCCTT
qRFT1-F	TGCCGGCCATGTCAAATTAATAAC
qUBQ-F	AACCAGCTGAGGCCCAAGA
qUBQ-R	ACGATTGATTTAACCAGTCCATGA
**Genotyping**	
Osgi-F1	CCCACAACTTATGCCATCCACG
Osgi-R1	CTGCCTTTTGTACGATGATACATAGCC
Osgi-GUS	TTGGGGTTTCTACAGGACGT
elf3-1-LP	AAGTGCGAATGGCAAAAGTC
elf3-1-RP	TTTCCTCTATGCCTCCTTGC
HPT-F	TACACAGCCATCGGTCCAGA
HPT-R	TAGGAGGGCGTGGATATGTC
ehd1-F1	GCCCTTGTATATATCTTGCATATGGT
ehd1-R1	GCTACTCCGGCTACCACGAGATCAAC
ehd1-F2	CAGAGGTGAGTAGGCTGACCACTAA
ehd1-R2	TTCTTCTAGATATATCTTACAAATATTCG
ehd1-F3	GTCTATCTGAGCGTATCTGAGCATG
ehd1-R3	GGAGTCAGTTATTAACTGTAGTTAGC
prr37-F1	GGACACACATCAGCATTTCATCCTGC
prr37-R1	GACAACTACTTCCAGTAACATGTTCTTTAG
se5-F1	CTACTCCTACATAATACCGTGTGCAATTTG
se5-R1	GTGTCCACACTATACAGTCAGGAAAGC

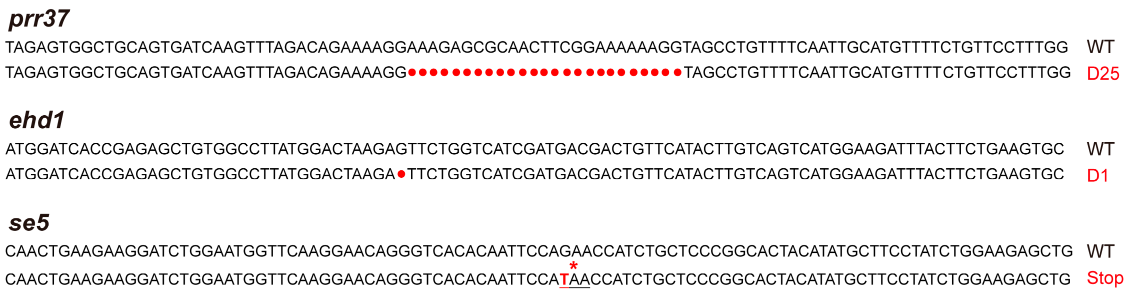

Figure S1. Sequence of different mutant alleles.

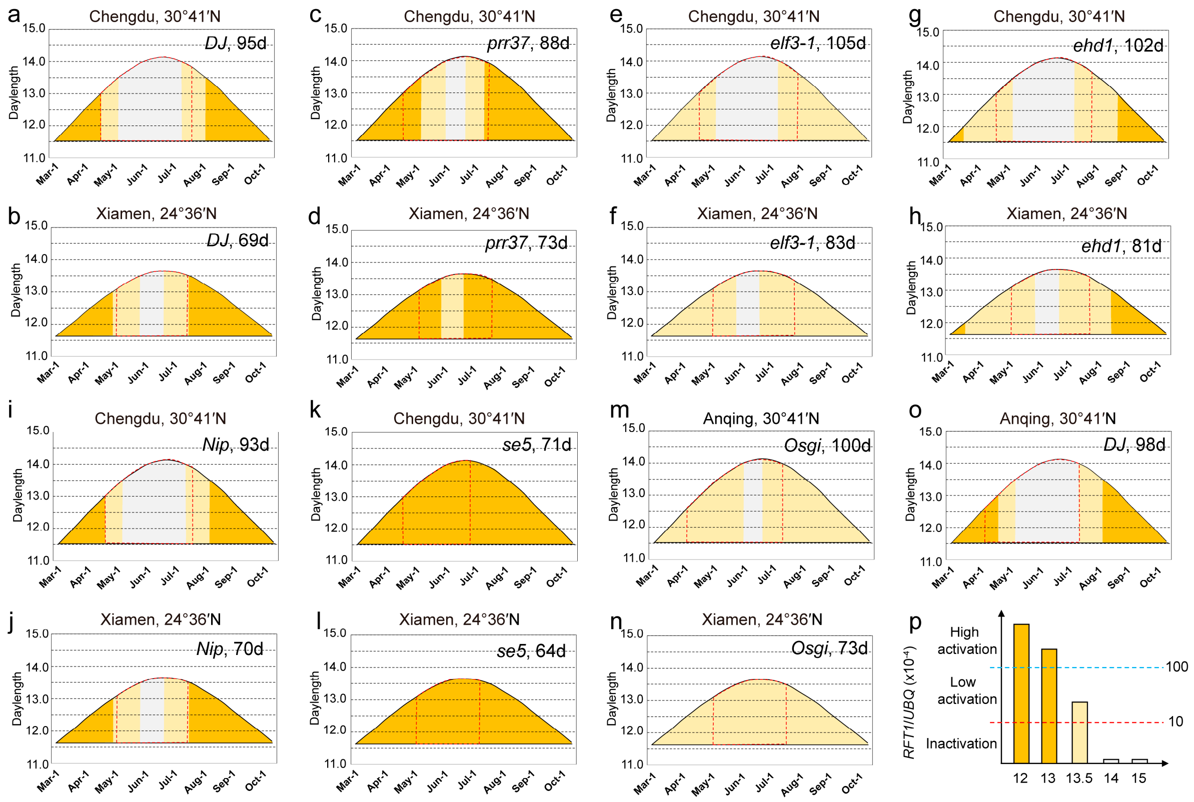

Figure S2. The effect of latitude and genotype on dynamics of *RFT1* transcript levels. **(a–l)** Predicted *RFT1* expression during the growing season in Xiamen (24°36′N) and Chengdu (30°41′N) and relation dynamics of *RFT1* transcription in response to different latitudes to flowering time in DJ **(a, b)**, *prr37* **(c, d)**, *elf3-1* **(e, f)**, *ehd1* **(g, h)**, Nip **(i, j)** and *se5* **(k, l)**. **(m–o)** Predicted *RFT1* expression of *Osgi* **(m, n)** and DJ **(o)** at Xiamen (24°36′N) and Anqing (30°41′N). **(p)** The three levels of *RFT1* gene-expression. The red dotted box represents the time from sowing to flowering. Flowering time is indicated at the top of each heatmap. Dark yellow, light yellow, and gray indicate high activation, low activation and inactivation of *RFT1* transcription, respectively.

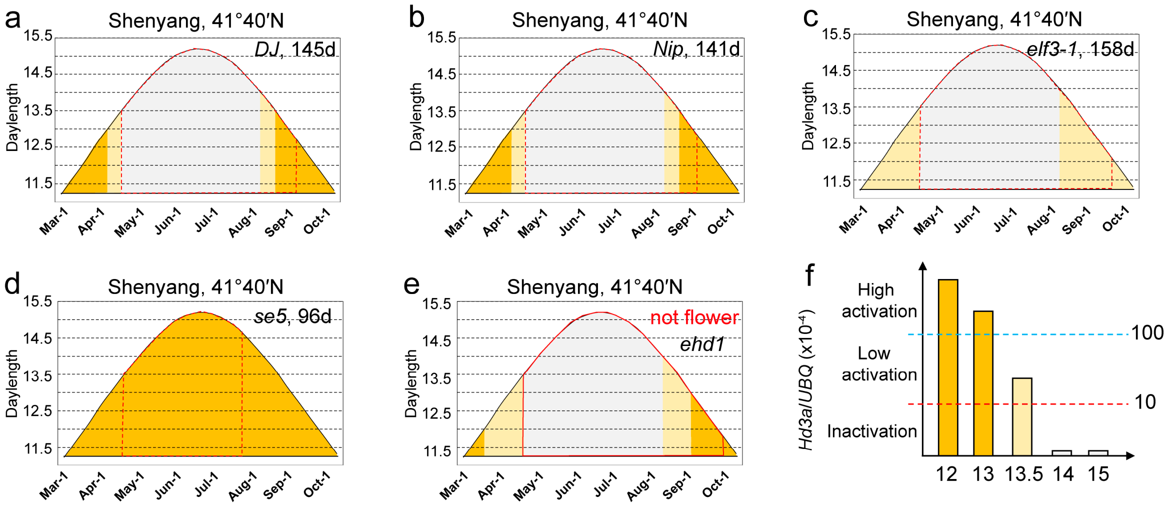

Figure S3. The effect of latitude and genotype on dynamics of *Hd3a* transcript levels. **(a–e)** Predicted *Hd3a* expression during the growing season in Shenyang (41°40′N) and relation dynamics of *Hd3a* transcription in response to 41°40′N to flowering time in DJ **(a)**, Nip **(b)**, *elf3-1* **(c)**, *se5* **(d)** and *ehd1* **(e)**. **(f)** The three levels of *Hd3a* gene-expression. The red dotted box represents the time from sowing to flowering. Flowering time is indicated at the top of each heatmap. Dark yellow, light yellow, and gray indicate high activation, low activation and inactivation of *Hd3a* transcription, respectively.

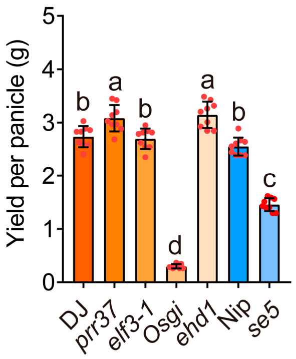

Figure S4. Yield per panicle of wild−type and mutant. Data are presented as means ± SD of nine biological replicates. Wild−type plants (DJ and Nip) and mutants are indicated at the bottom of each column. The letters above each column indicate significant differences by Duncan's multiple range test (P<0.05). The yield per panicle of wild−type and mutant were collected under natural conditions in Xiamen.
